# Treatment Outcomes of Advanced Stage Classical Hodgkin Lymphoma in the Setting of Vinblastine and Dacarbazine Shortages: A Case Series

**DOI:** 10.1155/crom/5446464

**Published:** 2026-04-03

**Authors:** Ishwarya Satyavarapu, Noah Giese, Prakruti Gandhi, Godsfavour Umoru, Ethan A. Burns, Sai Ravi Pingali

**Affiliations:** ^1^ Department of Medicine, Houston Methodist The Woodlands Hospital, Conroe, Texas, USA; ^2^ Department of Medicine, Houston Methodist Hospital, Houston, Texas, USA, houstonmethodist.org; ^3^ The University of Texas Cancer Center, Houston, Texas, USA, mdanderson.org; ^4^ Houston Methodist Neal Cancer Center, Houston Methodist Hospital, Houston, Texas, USA, houstonmethodist.org; ^5^ Department of Pharmacy, Houston Methodist Hospital, Houston, Texas, USA, houstonmethodist.org

## Abstract

**Purpose:**

Oncology drug shortages have significant implications on patient care, particularly with curative intent therapeutic strategies. This case series highlights strategic management for patients with classical Hodgkin lymphoma (cHL) in the setting of recent dacarbazine and vinblastine shortages without compromising outcomes.

**Summary:**

cHL is a highly curable malignancy with initial treatment strategies incorporating chemotherapy, radiation therapy, chemoradiotherapy, or chemoimmunotherapy. The standard of care for patients with cHL includes a backbone of doxorubicin, vinblastine, and dacarbazine (AVD) based therapies in combination with either bleomycin, brentuximab vedotin (BV), or nivolumab. However, in 2023, critical shortages of dacarbazine and vinblastine led to rationing of these agents, raising concerns for a paucity of clear management strategies in patients with cHL. The three patients described in this case series received lower cumulative doses of standard chemotherapy as opposed to alternative therapies without compromising outcomes.

**Conclusion:**

Treatment of advanced stage cHL during dacarbazine and vinblastine shortage requires careful consideration of alternative therapies and collaborative effort among healthcare professionals. This case series highlights that with a response‐added approach and vigilant monitoring, effective cancer treatment strategies may be implemented despite drug shortages.

**Summary**

• Oncology drug shortages should prompt the implementation of novel treatment approaches to minimize impact on clinical outcomes.
• In the setting of national drug shortages, providers can consider administering catch up doses of essential therapies at appropriate times during the patient’s treatment plan.
• Pharmacists play a critical role in managing drug shortages by collaborating closely with providers to anticipate operational needs while ensuring continuity and quality of patient care.

## 1. Introduction

Classical Hodgkin lymphoma is a highly curable malignancy with an overall survival rate greater than 88% at 5 years. Initial treatment strategies incorporate combinations of chemotherapy, radiation therapy, or chemoimmunotherapy for both early‐stage and advanced stage disease [1–3]. While standard‐of‐care regimens are clearly defined, outcomes and treatment strategies are not clearly established in cases of drug shortages. Oncology drug shortages may negatively impact patient morbidity and survival due to the utilization of suboptimal therapies [4]. The American Society of Clinical Oncology (ASCO) offers suggestions for alternative agents in the treatment of cHL; however, there is limited data on the efficacy of these regimens, and they are often based on consensus opinion rather than rigorously tested trials [5, 6].

Management of cHL is dependent on the disease stage, histologic subtype, and patient‐specific factors (e.g., age, performance status, and symptoms) [7]. Patients are classified as either early‐stage favorable (stage I or II with no unfavorable factors), early‐stage unfavorable (stage I or II with unfavorable factors), or advanced stage (stage III or IV) [2, 8]. Unfavorable factors of cHL include bulky lymphadenopathy, “B‐symptoms” (fever, drenching night sweats, weight loss), elevated erythrocyte sedimentation rate (ESR), and multiple involved nodal regions all of which are associated with a poorer prognosis and, therefore, guide treatment intensity [2]. The ABVD chemotherapy regimen (doxorubicin, bleomycin, vinblastine, and dacarbazine) remains the standard of care for management in patients with early‐stage cHL, with a positron emission tomography (PET)‐adapted approach guiding the use of additional cycles of therapy or radiation [9]. For stage III–IV disease, both BV‐AVD (brentuximab vedotin, doxorubicin, vinblastine, and dacarbazine) and nivolumab‐AVD have emerged as a preferred treatment option based on both options having a superior efficacy when compared to ABVD [10, 11]. Standard of care treatment includes 6 cycles of AVD based therapies with an interim PET after two cycles to further define therapy [2, 8, 9, 11].

A 2020 survey of 68 institutions reported vincristine and vinblastine were the two most difficult chemotherapeutic agents to obtain, with a further 67% of facilities reporting dacarbazine shortages in 2018–2019 [12]. In 2023, both dacarbazine and vinblastine experienced shortages due to a combination of increased demand and unspecified manufacturing delays which posed significant challenges to the treatment of cHL [13]. Omission of dacarbazine in the treatment of cHL is associated with significantly worse outcomes when not included in a treatment regimen. A 2015 noninferiority trial by the German Hodgkin Study Group (GHSG) HD13 studying dacarbazine omission was stopped early due to increased progression of disease [14]. When these shortages occur, there is some precedence for clinicians to guide decision‐making. PET‐CT scan can be used to evaluate response after the first two cycles of ABVD, and for those that demonstrate response, patients continue standard chemotherapy. For those that have aggressive or resistant disease, an escalated regimen (escBEACOPP) can improve 5‐year progression‐free survival (PFS) to 95.4% compared with 89.1% as demonstrated in the GHSG HD14 study, if procarbazine is available [15]. However, not all patients, particularly older patients or those with medical comorbidities, may be able to tolerate this regimen. Substituting medications or other adjustments to optimize treatment delivery must be considered by clinicians in the setting of a drug shortage, and there remains no clear guidelines on directing the best approach. It therefore remains of vital importance to seek ways to maintain patient outcomes despite a drug shortage. This case series highlights varying treatment strategies and outcomes for patients in their 30s to 40s with advanced stage cHL amidst an ongoing drug shortage of dacarbazine and vinblastine. Patient clinical characteristics are summarized in Table 1.

**Table 1:** Clinical characteristics, treatment modifications, and patient outcomes due to drug shortages.

Case	Age	Sex	Histological subtype	Stage	Treatment regimen	Doses missed	Additional treatment	CR duration	Interim PET best response
1	30s	M	Not otherwise specified (nodular lymphoid architecture)	III	BV‐AVD	1 dose dacarbazine3 doses vinblastine	1 dose vinblastine and dacarbazine after cycle 6	24 months	Complete metabolic response, Deauville score of 2
2	40s	M	Mixed cellularity	IV	BV‐AVD	1 dose dacarbazine3 doses vinblastine	1 dose vinblastine and dacarbazine after Cycle 6	10 months	Complete Metabolic Response, Deauville score of 2
3	20s	F	Not otherwise specified (EBV‐associated)	IV	BV‐AVD with substitutions(vinblastine → vincristine; dacarbazine shortage managed with BV + AV)	4 doses dacarbazine4 doses vinblastine	No additional treatment	13 months	Complete metabolic response, Deauville score of 1

## 2. Case Series

### 2.1. Case 1

A male in his fourth decade of life with no significant past medical history presented with weight loss, extreme fatigue, poor appetite, intermittent fevers, and lower back pain unresponsive to over‐the‐counter nonsteroidal anti‐inflammatory drugs. Computed tomography (CT) of the chest, abdomen, and pelvis revealed multiple large bulky right axillary and subpectoral lymph nodes, with the largest measuring up to 4.8 cm. A biopsy of the right axillary lymph node was consistent with cHL, with a few scattered large Epstein–Barr virus (EBV)‐positive cells; the subtype was not otherwise specified, although nodular lymphoid architecture without fibrosis was noted. A bone marrow biopsy was subsequently performed, which confirmed no morphologic evidence of cHL.

PET/CT imaging confirmed active lymphoma within the right chest and spleen, establishing stage III disease. Treatment with BV‐AVD was initiated, and an interim PET/CT scan after three cycles showed complete metabolic response with a Deauville score of 2. During the final three cycles of BV‐AVD, the patient failed to receive one dose of dacarbazine and three doses of vinblastine due to an ongoing national drug shortage. Although the restaging PET/CT scan confirmed no evidence of active lymphoma, the treating hematologist decided to administer one dose of vinblastine and dacarbazine after completion of six cycles to make up for missed doses. At last follow‐up, 24 months after completion of therapy, the patient remains in complete response (CR).

### 2.2. Case 2

A male in his fifth decade of life with previously untreated cHL, mixed cellularity subtype, presented with stage IIB disease. ABVD was recommended, but he refused therapy and was lost to follow‐up for 10 months. He presented again with progressive fatigue, shortness of breath, adenopathy, and bilateral pulmonary infiltrates indicative of progressive disease, restaged as having stage IV disease. PET/CT was suggestive of bulky disease in the chest, with evidence of a large pericardial effusion. Bone marrow biopsy confirmed no morphologic involvement by cHL.

Given findings consistent with advanced‐stage disease, treatment with BV‐AVD was initiated. After two cycles of BV‐AVD, an interim PET showed a complete metabolic response, with a Deauville score of 2. During the final three cycles of BV‐AVD, the patient failed to receive one dose of dacarbazine and three doses of vinblastine. A restaging FDG‐PET scan after completion of six cycles again demonstrated a complete metabolic response with a Deauville score of 2. Similar to Case 1, the treating hematologist elected to administer one dose of vinblastine and dacarbazine after cycle six to make up for missed doses. At 10 months following completion of therapy, the patient remains in CR, with stable findings seen on the most recent PET/CT.

### 2.3. Case 3

A female in her fourth decade of life with no significant past medical history presented to the emergency department with 1 month of fevers and lymphadenopathy. CT imaging demonstrated left cervical, retroperitoneal, and pelvic lymphadenopathy. A cervical lymph node core biopsy was positive for EBV‐associated cHL, subtype not otherwise specified. Given elevated liver function tests, a liver biopsy was also performed, which confirmed hepatic cHL involvement. Bone marrow biopsy showed no evidence of involvement.

Cycle 1 treatment consisted of dose‐adjusted BV‐AVD due to persistent fevers and concern for hemophagocytic lymphohistiocytosis (HLH). During cycle 2, there was a national shortage of both vinblastine and dacarbazine. Vinblastine was substituted with vincristine; however, the patient opted for no change in regimen for the dacarbazine shortage, thus receiving BV plus AV. During cycle 3, due to a continued dacarbazine shortage, the regimen was modified to BV‐CHP.

An interim PET/CT scan after cycle 3 demonstrated no evidence of active lymphoma, with a Deauville score of 1. The patient completed the remaining three cycles with the standard chemotherapy regimen, BV‐AVD. No additional doses of the missed vinblastine or dacarbazine were administered after completion of six cycles. Liver function tests and EBV levels normalized with treatment, and complete clinical, morphologic, and radiographic remission were achieved [16]. In contrast to Cases 1 and 2, in which missed doses were made up after completion of therapy, missed doses in Case 3 were substituted with alternative agents. Given the sustained complete metabolic response, normalization of laboratory abnormalities, and interval imaging demonstrating a Deauville score of 1, continued observation was considered reasonable, thereby avoiding the potential risk of additive toxicities with limited or uncertain benefit. At 13 months following completion of therapy, the patient remains in CR.

## 3. Discussion

The primary goal of treatment for patients with cHL is to achieve durable remission while minimizing long‐term toxicities [17]. The aim of this paper is to demonstrate that effective treatment of advanced stage cHL is possible in the setting of national drug shortages with vigilant monitoring and alternative treatment strategies. In Cases 1 and 2, interim PET‐adapted imaging after cycle three and two of BV‐AVD therapy, respectively, showed complete metabolic response. Patients continued BV‐AVD treatment for the remaining cycles; however, instead of drug substitutions, they instead received “make‐up doses.” Case 3, however, experienced chemotherapy agent shortages early on in treatment. The patient received drug substitutions and later received recommended therapy when the chemotherapy became available. These three cases highlight that despite drug shortages, PET‐adapted imaging helps identify disease status, allowing clinicians to prioritize available chemotherapy and aid in decision‐making strategies. This approach is supported by National Comprehensive Cancer Network guidelines, which recommend two cycles of ABVD for all early‐stage cHL patients and either BV‐AVD or nivolumab‐AVD for all advanced stage cHL patients as category one recommendations [2, 10]. An integrated PET/CT with the incorporation of the Deauville 5‐point scale score is recommended to assess interim response after two cycles to guide subsequent treatment selection [18, 19]. There has also been research using PET/CT to direct treatment decisions including de‐escalating bleomycin based on PET/CT progression. However, the authors noted that omission of bleomycin did not meet the cut‐off of noninferiority but had similar efficacy and patients had reduction in pulmonary toxicities [19].

In recent decades, combined modality treatment (CMT) with chemotherapy and involved site radiation therapy (ISRT) has replaced radiation therapy alone as the standard of care due to potential toxicities associated with high‐dose, large‐field irradiation [20, 21]. The GHSG conducted a multicenter HD10 trial to investigate the relative intensity of chemotherapy and involved field radiation therapy (IFRT) in 1370 patients with newly diagnosed, early‐stage favorable HL [22]. Patients were randomly assigned to receive four cycles of ABVD followed by either 20 or 30 Gy of IFRT or two cycles of ABVD followed by either 20 or 30 Gy of IFRT. There were no significant differences in overall survival (OS; 97.1% vs. 96.6%), PFS (93.5% and 91.2%), and freedom from treatment failure (FFTF; 93.0% vs. 91.1%) when four cycles of ABVD were compared against two cycles of ABVD. The final analysis confirmed that two cycles of ABVD with 20 Gy of IFRT was an effective and less toxic treatment option for patients with early‐stage favorable disease [22]. Therefore, during a drug shortage, one strategy employed by clinicians is to prioritize and ration available chemotherapy to patients with improved PET avidity [15]. Following the data from HD10 published by the GHSG, those with favorable response can be given a reduced intensity regimen, thus increasing the number of patients who can benefit from a limited quantity of chemotherapeutic agent [22, 23].

Incorporation of CMT is a cornerstone in the management of cHL given the high response and durable remission rates. Vinblastine is a critical component in CMT regimens due to its demonstrated effectiveness as part of a multidrug chemotherapy regimen. The vinblastine shortage had significant implications on the overall management of cHL [13]. Oncologists primarily modified standard protocols by either delaying treatment, omitting vinblastine, or substituting therapies. The hematologist at our institution achieved comparable response rates with omission of vinblastine during the treatment cycles and subsequent make‐up after Cycle 6 of treatment.

## 4. Best Practices During Drug Shortages

Several strategies can be implemented to optimally manage limited supply during periods of drug shortages. Dose rounding of cytotoxic chemotherapy to the nearest vial size is practiced among many healthcare institutions to reduce healthcare costs and minimize drug wastage [24, 25]. The Hematology/Oncology Pharmacy Association (HOPA) states that dose changes ≤10*%* are not expected to reduce the safety or efficacy of therapy when considered within the context of standard dose adjustments for tolerance and tumor response, clinical trial deficiency criteria, and the influence of interpatient pharmacokinetic availability [26]. As such, our institution considers it reasonable to adjust doses of cytotoxic chemotherapy agents within 5% of the calculated dose. During the nationwide vinblastine shortage, NCCN guidelines recommended capping the vinblastine dose at 10 mg to avoid wasting an entire vial since vinblastine is only available in a 10mg vial [2].

Treatment plan modifications can be considered by temporarily substituting vinblastine with vincristine or BV‐AVD with BV‐CHP (brentuximab vedotin, cyclophosphamide, doxorubicin, and prednisone). Regimens containing vincristine, such as BEACOPP (bleomycin, etoposide, doxorubicin, cyclophosphamide, vincristine, procarbazine, and prednisone), have demonstrated efficacy as subsequent line treatment options for cHL [6, 27]. In a meta‐analysis by Skoetz et al., there was moderate‐ to high‐quality evidence that escalated BEACOPP provided PFS and OS benefit in early‐stage unfavorable and advanced stage cHL patients [27]. However, escalated BEACOPP was found to be more toxic with a higher risk for long‐term toxicities when compared to the standard ABVD [6]. To our knowledge, there have been no randomized controlled trials evaluating the efficacy of vincristine in place of vinblastine in the standard ABVD regimen, limiting our ability to ensure similar treatment outcomes.

Additionally, pharmacists play a pivotal role in healthcare systems by ensuring continuity and quality of patient care despite the challenges posed by global drug shortages. Pharmacists are responsible for collaborating closely with members of the healthcare team to communicate shortages, identify effective alternatives, adjust treatment regimens, and educate patients about treatment plan modifications [28]. They play a critical role in inventory management by anticipating operational needs while minimizing disruptions in patient care. Additionally, pharmacy leaders contribute to staff training, ensuring that healthcare providers are equipped to manage shortages effectively through ongoing education and readiness to adapt to evolving circumstances in patient treatment [29].

## 5. Suggested Treatment Approach

Oncology drug shortages should prompt the implementation of novel treatment approaches to minimize impact on clinical outcomes. In the setting of national drug shortages, providers can consider administering catch up doses of essential therapies at appropriate times during the patient′s treatment plan. Alternative regimens for vinblastine shortages include substituting vincristine or BV‐CHP. If a patient is anticipated to tolerate toxicities, escalated BEACOPP can be considered especially if the drug delay is anticipated to be extensive. Pharmacists play a critical role in managing drug shortages by collaborating closely with providers to anticipate operational needs while ensuring continuity and quality of patient care.

## 6. Conclusion

Management of cHL often involves initial treatment with ABVD or BV‐AVD with or without radiation therapy. Treatment of cHL during a vinblastine shortage requires careful consideration of alternative therapies and collaborative effort among healthcare professionals. This case demonstrates that with novel approaches and vigilant monitoring, effective cancer treatment can still be achieved despite drug shortages.

## Funding

No funding was received for this manuscript.

## Disclosure

At the time of writing, Prakruti Gandhi was affiliated with the Houston Methodist Hospital in Houston, Texas.

## Conflicts of Interest

The authors declare no conflicts of interest.

## Data Availability

Data sharing is not applicable to this article as no datasets were generated or analyzed during the current study.
